# Age-Related Dichotic Listening Skills in Impaired and Non-Impaired Readers: A Comparative Study

**DOI:** 10.3390/jcm12020666

**Published:** 2023-01-14

**Authors:** Pierre Reynard, Charles-Alexandre Joly, Maxime Damien, Marie-Thérèse Le Normand, Evelyne Veuillet, Hung Thai-Van

**Affiliations:** 1 Institut de l’Audition, Institut Pasteur, INSERM U1120, 75012 Paris, France; 2 Faculty of Medicine, University Claude Bernard Lyon 1, 69100 Villeurbanne, France; 3 Service d’Audiologie et d’Explorations Oto-Neurologiques, Hôpital Edouard Herriot, Hospices Civils de Lyon, 69003 Lyon, France; 4 Laboratoire de Psychopathologie et Processus de Santé, Université de Paris Cité, 92100 Boulogne-Billancourt, France

**Keywords:** impaired readers, non-impaired readers, auditory processing disorder, dichotic listening, binaural integration, binaural separation, free recall, directed attention, intrusive response

## Abstract

Dichotic listening is the high-level auditory process which enables the perception of different verbal stimuli delivered simultaneously to the right and left ears (binaural integration), as well as the perception of a verbal stimulus presented to one ear while ignoring a different stimulus in the other ear (binaural separation). Deficits in central auditory processing have been reported in children with learning disabilities. The present study aimed to compare dichotic listening performances in right-handed impaired readers (IR) and non-impaired readers (non-IR) according to age. For this, a cross-sectional study was conducted in 120 IR (56 males and 64 females) divided into five age groups and 120 non-IR (63 male and 57 female) matched on chronological age (8 to 9 years; 9 to 10 years; 10 to 12 years; 12 to 18 years; adult). They were tested for binaural integration and binaural separation, allowing for the calculation of dichotic aptitude (DA), ear prevalence (EP), and attentional shift index (ASI). A series of ANOVAs showed an effect of age and of the reading group for all the dichotic-related measures, except for EP. Binaural separation scores were lower in IR who also showed more intrusive responses compared to non-IR. These intrusive responses, which were more frequent on the right ear for IR, decreased with age in both groups. Overall, these results suggest that dichotic listening scores improve with age as the central auditory pathways mature. However, whatever the age, performances are lower in IR than in non-IR. This might be explained by an incomplete maturation of the auditory pathways in IR; an early start for long-term follow-up and auditory training is suggested.

## 1. Introduction

Dichotic listening (DL) paradigms have been used extensively as non-invasive behavioral procedures to assess language lateralization among children with and without learning disabilities and in individuals suffering from other auditory system-related brain disorders [1]. DL tests correspond to the simultaneous presentation of different acoustic signals in both ears [2]. The participant is asked to verbally reproduce either all the perceived signals (free recall) or only those perceived in one ear (directed attention). Attention plays an important part in DL tests, and is considered as a source of variance in DL results [3,4]. Attentional manipulations were used in dichotic listening studies to control for attentional bias; Asbjornsen et al. proposed an index of attentional shift (The Attentional Shift Index (ASI)) based on the ratio of correct responses and intrusion errors when the subject is performing the tasks of directed attention [4]. DL tests allow us to measure both binaural integration and separation, which are crucial skills for speech sound extraction in the presence of concurrent auditory information, as well as to measure the integrity of cerebral hemispheres and the corpus callosum (CC) [5,6,7]. Due to the highly myelinated nature of its fibers, the CC provides a means of interhemispheric communication at very fast pulse rates [8]. The posterior part of the CC (isthmus) may be involved in auditory information transfer [8] and could be a primary component of dichotic processing. The anterior commissure, located posterior to the rostrum of the CC, has also been suspected of harboring auditory fibers, but in an accessory manner [5]. 

The right ear advantage (REA) is the fundamental notion of dichotic listening, originally described by Kimura [9,10]; it is described as an outperformance of the right ear (RE) over the left ear (LE) [10,11,12]. REA is seen in 90% of healthy children and adults [13]. A REA prevalence of 80–85% in right-handed adults and 50% in left-handed adults was reported [14]. Left ear advantage (LEA) is most often found in typically developing listeners for non-verbal stimuli or emotional tone of verbal stimuli [15,16]. 

The maturation of DL appears to be related to the maturation of the CC. Impaired communication between the two hemispheres would contribute to an increased LE deficit or increased REA, and extinction of LE reports from the temporal lobe [6]; such impairment via a CC lesion has been described in many clinical cases [7]. Because CC maturation occurs in the first two decades of life, it seems likely that improvement in dichotic performances may be related to that maturation [7,17]. Neuroimaging and behavioral studies suggest the existence of a critical time period for CC functional development, which lies between the ages of 6 and 8 [17,18]. Furthermore, according to the American Academy of Audiology, normative ranges for the majority of behavioral tests in very young children (<7 years old) have limited clinical utility, due to very large standard deviations and resultant floor or chance effects (https://audiology-web.s3.amazonaws.com/migrated/CAPD%20Guidelines%208-2010.pdf_539952af956c79.73897613.pdf, accessed on 14 May 2021). 

Children with reading impairments may show slower lateralization development compared to typical children, or this development may not take place at all. Whether this atypical development is a cause, correlate, or consequence of language development is unknown [19]. Impaired readers (IR) have long been associated with problems in central auditory processing abilities and with atypical cerebral lateralization. Although reading classically activates left hemisphere networks for speech processing [20], IR could show less activated networks compared to typical readers with heightened right hemisphere activation during reading or other phonological tasks [21].

Developmental dyslexia is a common neurodevelopmental disorder related to a difficulty in automatically associating graphemes and phonemes and could affect up to 10% of children [22,23]. Efficient automatic reading requires visual analysis of the graphemes and their association with phonemes and/or with larger phonological units [24], and efficient temporal processing [25]. Many other auditory processing deficits may explain poor phonological skills in developmental dyslexia [26]. In typical readers, language lateralization develops from diffuse hemispheric representation in infancy to left hemispheric lateralization in adulthood [27], and disruption of this lateral development could lead to problems with language and literacy [19]. Disrupted performances were found considering DL performances in children with reading difficulties [28]. 

Concerning ear prevalence (EP, i.e., interaural asymmetry in favor of the RE or the LE), two main patterns have been described in IR and dyslexic individuals. The first one reports a strong interaural asymmetry in favor of the RE, explained by a reduced performance in the LE or a low LE report with a right hemisphere under-engagement [29,30,31], as is classically described in children with central hearing impairment [32,33]. This suggests the existence of impaired inter-hemispheric transfer in accordance with Kimura’s model [9,10]. Other studies, using consonant and vowel syllables as stimuli, report symmetrical performance in dyslexic children with a much lower REA [34] and even a RE deficit [35]. This weakness of the RE would persist during adolescence and would still be present in adulthood [13]. Finally, other studies report an advantage of the LE in dyslexic children during the free recall task, suggesting the possibility of a more developed planum temporale on the right [36]. Using digits and words, Demanez et al. reported significantly lower DL performances (correct responses on both ears) in a sample of 83 12-year-old dyslexic children when compared to their peers [37]. To help in the clinical assessment of children suspected of having an auditory processing disorder, Moncrieff et al. developed normative dichotic performance values from 416 typically developing children aged 5 to 12 years [38]. A REA was found in nearly 60% of these children.

All previously published studies show poor DL performances in IR and children with dyslexia of various age levels; however, there are no standard values defined in the literature for IR. It can be hypothesized that there is an increase in DL performances in IR with age, but that the latter do not catch up to the performances of non-IR. The objective of the present study was to compare DL performances between IR and non-IR according to age, from age 8 years old (i.e., the earliest age when the diagnosis of IR can be established) to adulthood.

## 2. Materials and Methods

### 2.1. Participants

This cross-sectional study was conducted with 240 native French-speaking participants, divided into two reading groups (IR vs non-IR) and of five age levels (8 to 9 years; 9 to 10 years; 10 to 12 years; 12 to 18 years; adult). Details of the total number of individuals in each group, individuals in each gender for each group, and mean age and SD for each group are shown in Table 1.

The experiment was carried out with the approval of the regional ethics committee (CPP Sud-Est IV, Lyon, France; approval no. 09/086 for adults and 04/008 for children). Informed consent was obtained from all participants (children’s parents, as well as a verbal consent from all children) prior to participation.

Inclusion criteria were air conduction thresholds less than 15 dB in the octave frequencies 250 Hz to 8 kHz; normal middle ear functioning was confirmed by type A tympanograms with ipsilateral and contralateral acoustic reflexes present for the frequencies 500 Hz, 1 kHz, and 2 kHz. IR was diagnosed according to the Diagnostic and Statistical Manual of Mental Disorders fourth edition (DSM IV), with a reading level at least 18 months behind the norm. Reading age was assessed using the French reading test “L’alouette”, which evaluates reading level in terms of word and non-word decoding and reading speed [39]. All patients were right-handed with a laterality quotient ≥71.4%. To better control for effects of handedness that may shift the direction of ear advantage, the degree of handedness was assessed using the Edinburgh Handedness Inventory [40], and only right-handed children were included. Exclusion criteria were psychopathological disorder and neurological impairment.

### 2.2. Dichotic Listening Assessment

The procedure described by Demanez et al., which comprises lists from a French central auditory processing assessment battery [41] and was designed based on the American Speech-Language-Hearing Association Consensus Statement on central auditory processing and models [42], was used. The material is made of 50 pairs of dichotic stimuli divided into five lists of 10 items. In each list, the verbal stimuli are grouped in pairs (words, digits, syllables) that are balanced acoustically, phonetically, linguistically, and semantically. The dichotic paradigm can be performed in two conditions, both obtained with stimuli presented in both ears. The first (free recall condition) was obtained when a participant was asked to repeat what he heard in both the RE and LE ( = undesignated ears). In this condition, binaural integration and divided attention are studied. The second (directed or forced recall condition) was obtained when the participant was asked to repeat stimuli presented in a designated ear and to ignore the information delivered in the other ear (attended right ear (AR) or attended left ear (AL)). In this second condition, binaural separation and directed attention were studied. For each participant, the lists were presented in the following order: list of words (substantive) in free recall condition (example: “*navet*” = turnip and “*rideau*” = curtain); list of bisyllabic words (substantive) in directed recall condition (example: “*gamin*” = kid and “*ballon*” = ball); list of two digits in free recall condition (example: “9–6” and “10–7”); list of three digits in directed recall condition (example: “9–5–6” and “6–8–10”); list of two monosyllabic words (adjectives) in free recall condition (example “*mur-cher*” = ripe- expensive and “*riche-sage*” = rich-wise).

Overall, the protocol consists of three free recall and two directed recall tasks, administered alternately but in the same order for all participants. For directed recall conditions, the designated ear was changed after the administration of groups of five pairs. A response is considered complete ( = 1 point) when all the items perceived on the RE and on the LE are entirely reproduced (free recall condition or when all the items perceived by the designated ear are entirely reproduced (directed recall condition). The set of subtests provides a total out of 50 (for each complete response), to be multiplied by two to get a result out of 100. Stimuli were presented via THD39 earphones (Telephonics, Farmingdale, NY 11735, USA) at an intensity of 60 dB SPL. 

### 2.3. Dichotic Listening Scores

Several indices were computed to assess DL performances. The dichotic aptitude (DA) is the sum of all complete responses in all conditions. It quantifies how well a participant identifies items presented to each ear, simultaneously. EP is the subtraction of right and left complete responses, divided by the total of complete responses and multiplied by 100; the result is a percentage.

As attentional factors could lead to a bias and can be evaluated by assessing intrusive responses [4,43], the attentional shift index (ASI) was calculated for directed recall tasks. It is the ratio of correct responses over intrusive errors in both ears: ASI = ln [REAR * LEAL)/(LEAR * REAL)]. For instance, LEAR represents the number of left intrusive responses, i.e., the number of stimuli presented to the LE that were repeated by the participant while being asked to focus on the RE.

Finally, the scores collected during the free recall tests were used to calculate a response score per ear (RE score and LE score), thus allowing another way of estimating ear prevalence by directly analyzing the preferential use of a particular ear for listening.

### 2.4. Data Analysis

Statistical analyses were conducted using two different kinds of analysis of variance (ANOVA). The first analysis was based on the design “5 age levels × 2 groups” with separate ANOVAs for each index (i.e., DA, EP, and ASI). The second analysis was based on the design “5 age levels × 2 groups × 2 ears”, with separate ANOVAs for each condition (directed or forced condition vs free recall condition). Significance level was set at 0.05. All analyses were run using Jamovi software (https://www.jamovi.org/download.html, accessed on 14 May 2022).

## 3. Results

Among the 240 children included (IR = 120 and non-IR = 120), the number of female (*n* = 64) and males (*n* = 56) in the IR group were balanced, and children were equally divided into the five age levels of 24 participants each. This sample of IR was matched for age to a non-IR group (63 male, 57 female), including the same five age levels (*n* = 24; Table 1).

### 3.1. Dichotic Aptitude and Ear Prevalence

**Figure 1:**
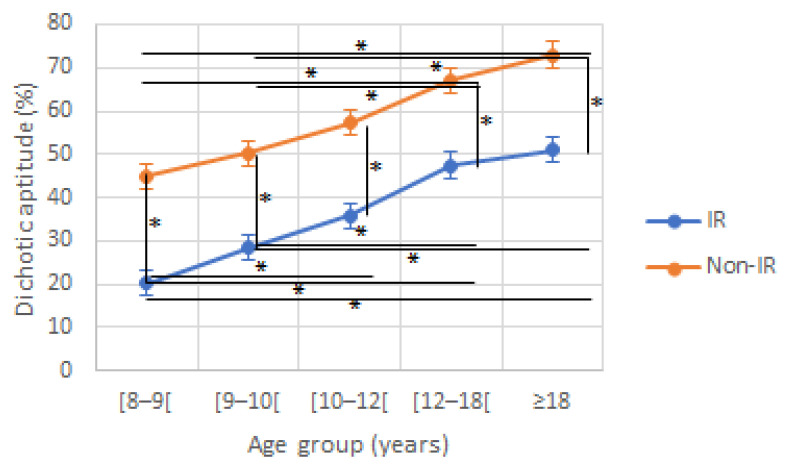
Dichotic aptitude (DA) according to age and reading group. IR: impaired reader; Non-IR: non-impaired reader. * indicate a significant difference between two values.

Regarding DA, a significant effect of age (F (4230) = 27.30, *p* < 0.001) and reading group (F(1230) = 119.53, *p* < 0.001) was found without any significant interaction effect (F(4230) = 0.746, *p* = 0.562; Figure 1). DA increased with age, whatever the reading level, but IR presented scores that were significantly lower than in non-IR for all age groups.

**Figure 2:**
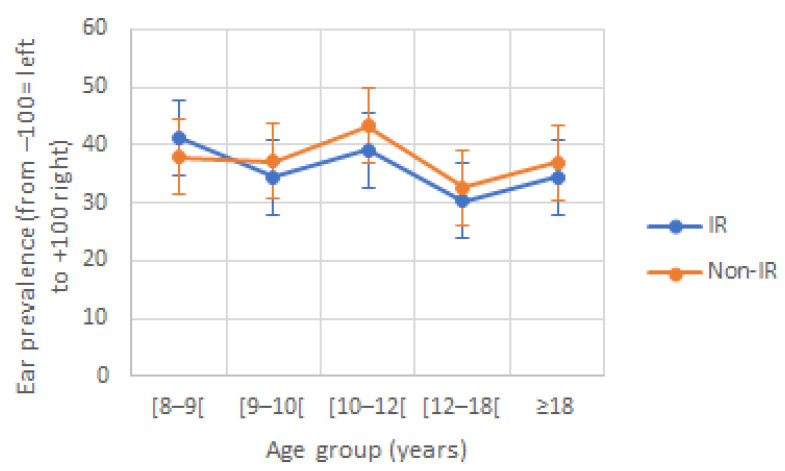
Ear prevalence (EP) according to age and reading group. IR: impaired reader; Non-IR: non-impaired reader.

Regarding EP, there was a REA in both reading groups. No significant effect of age (F(4230) = 0.683, *p* = 0.605) nor reading group (F(1230) = 0.170, *p* = 0.681) was observed, and there was no significant interaction effect (F(4230) = 0.104, *p* = 0.981; Figure 2).

### 3.2. Attended Ear in Directed or Forced—Recall Condition

**Figure 3:**
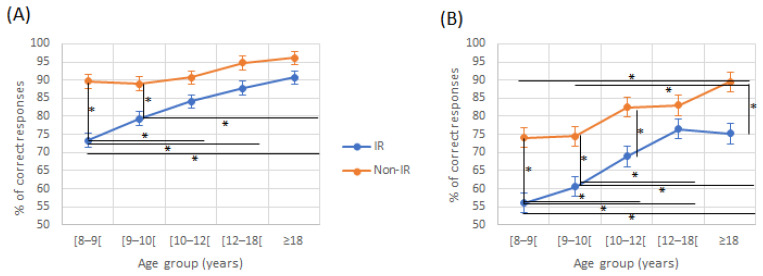
Attended Right (**A**) and left (**B**) scores in directed recall condition according to age and reading group. IR: impaired reader; Non-IR: non-impaired reader. * indicate a significant difference between two values.

There was a tendency for fewer correct responses for both ears on the attended ear, in the IR compared to non-IR. This difference was significant for certain age groups (Figure 3). There were also fewer correct responses on the Attended Left (AL) condition in both reading groups, this difference being significant for certain age groups (Table 2). For correct responses on the RE in the Attended Right (AR) condition, there was an effect of age (*p* < 0.001) and the reading group (*p* < 0.001). In the AR condition, improvement was higher in the IR group, as there was an interaction effect (F(4230) = 2.67, *p* = 0.003). In the AL condition, there was an effect of age (F(4230) = 15.11, *p* < 0.001) and of the reading group (F(4230) = 58.31, *p* < 0.001) with no interaction effect (F(4230) = 1.17, *p* = 0.325).

**Figure 4:**
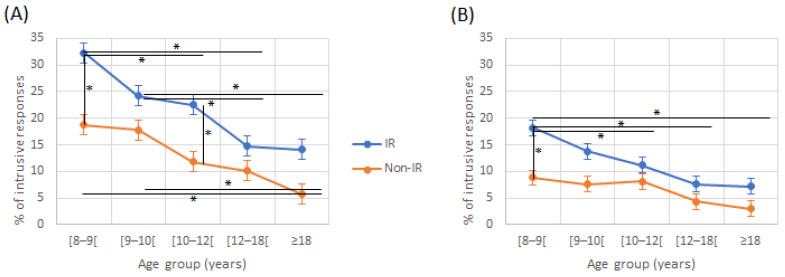
Intrusive responses from the right (**A**) and left (**B**) ears in directed recall condition according to age and reading group. IR: impaired reader; Non-IR: non-impaired reader. * indicate a significant difference between two values.

The percentage of intrusive responses decreased significantly with age for IR in the AR condition and in non-IR in the AL condition. This decrease was more pronounced for the RE in both reading groups. The difference between LE and RE intrusive responses was significant only for 8–9-, 9–10-, and 10–12-year-old children in the IR group, and for 8–9-and 9–10-year-old children in the non-IR group (Figure 4 and Table 2). For intrusive responses from the RE in the AL condition, there was an effect of age (F(4230) = 21.89, *p* < 0.001) and of the reading group (F(1230) = 32.21, *p* < 0.001), with no interaction effect (F(4230) = 1.71, *p* = 0.149). For intrusive responses from the LE in the AR condition, there was an effect of age (F(4230) = 11.28, *p* < 0.001) and of the reading group (F(1230) = 32.21, *p* < 0.001), with no interaction effect (F(4230) = 1.6, *p* = 0.175; Figure 4).

### 3.3. Correct Responses for the Right and Left Ears in the Free Recall Condition

Scores were higher for non-IR compared to IR, and there was a tendency towards higher scores on the RE for both reading groups (Table 2). An effect of age (F(4230) = 18.06, *p*.< 0.001) and of reading group (F(1230) = 92.63, *p* < 0.001) was found for the RE. 

Similar patterns were found for the LE, with an age effect (F(4230) = 21.13, *p* < 0.001) and a reading effect (F(1230) = 79.58, *p* < 0.001).

**Figure 5:**
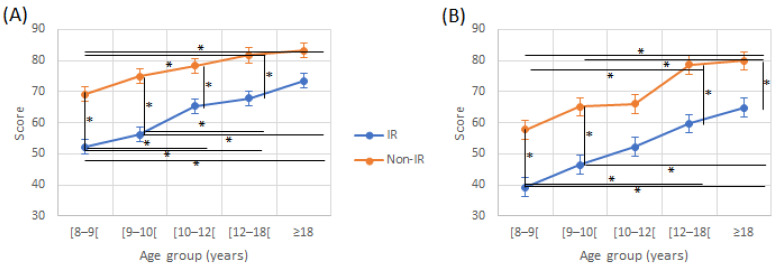
Right (**A**) and left (**B**) ear scores in free recall condition according to age and reading group. IR: impaired reader; Non-IR: non-impaired reader. * indicate a significant difference between two values.

No interaction effect was found for both ears (Figure 5).

### 3.4. Attentional Shift Index

**Figure 6:**
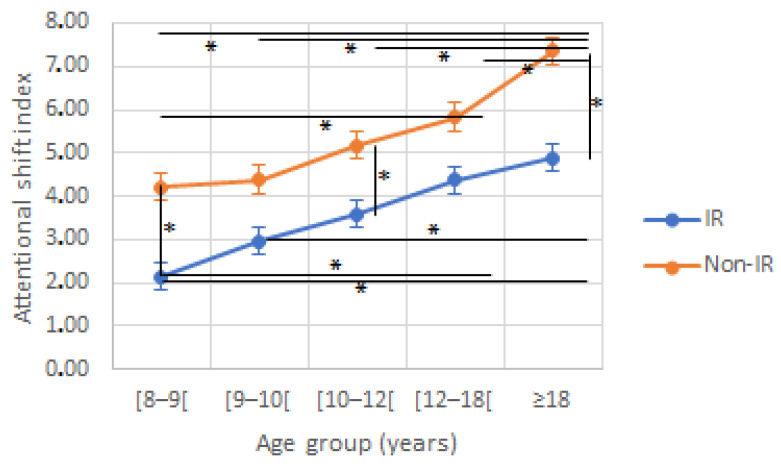
Attentional shift index according to age and reading group. IR: impaired reader; Non-IR: non-impaired reader. * indicate a significant difference between two values.

ASI was lower for the IR compared to the non-IR, a difference which was significant for the 8–9–, 10–12–, and ≥18-year-old children. There was an effect of age and reading group (F(4230) = 25.912 and F(1230) = 194.42, respectively, *p* < 0.001 for both) on ASI, with no interaction effect (F(4230) = 0.992, *p* = 0.413; Figure 6).

## 4. Discussion

The results of the present study show a gradual improvement in dichotic performances with age in both non-IR and IR. Scores in DA, AR, and AL improved in both groups, while intrusive responses in both ears decreased. ASI also improved with age in both groups. Although there was a similar improvement in non-IR and IR according to age, IR showed lower DL performances than non-IR at all ages, except for AR responses, for which an interaction between age and reading group was found. For both reading groups, EP showed a REA, with no significant difference between the two groups. Overall, these results confirm previous studies showing that dichotic processing improves with age [7]. As the CC may be a primary component of dichotic processing [8], there are reasons to believe that its maturation is necessary to improve dichotic performances during childhood development. 

The percentage of correct responses in the directed recall condition (binaural separation) was found to be lower in IR than in non-IR, with an increase in intrusive responses. Similarly to what was observed in non-IR, intrusion errors decreased with age in IR. However, more errors were found for the RE in the IR group. In the present study, despite an improvement in performances with age, DA in IR remained lower than the DA of non-IR of the same age, which is in agreement with several studies reporting poor dichotic performances in right-handed IR [13,34,44]. For example, Moncrieff et al. compared two groups (dyslexic and controls) of right-handed 11-year-old children. Dyslexic children performed more poorly than controls from their LE when listening to digits and words and from their RE when listening to consonants and vowels [35].

Such a difference between the DA of non-IR and IR groups may have several explanations, such as damage to or a maturation deficit of the CC or other brain regions implicated in auditory perceptual processing. In a magnetic resonance imaging (MRI) study, Hynd et al. compared the CC volume between dyslexic and matched control children and found the anterior region of the CC to be significantly smaller in the dyslexic children [45]. Other anatomical studies have shown differences in the development of CC fibers between dyslexic and typical reading children, supporting the hypothesis of less myelination in an immature brain [46,47,48]. In another MRI study, Casanova et al. showed that dyslexic patients had a reduced brain volume and decreased gyrification, but increased CC volume [49]. More recently, Elnakib et al. proposed introducing brain MRI as a supplement for dyslexia diagnosis, to quantify the anatomical differences in the CC of dyslexic and control subjects [50]. The study revealed significant size differences between 14 controls and 16 dyslexic participants in all four anatomical divisions of their CC. Besides CC modifications, anatomical studies have shown that other regions of the brain specific to auditory perceptual processing differ in dyslexic subjects, including the planum temporale [36] and medial geniculate nuclei [51,52]. 

Another explanation to the observed differences in DA performances between reading groups could relate to the methodology used herein. From a clinical point of view, DL tests provide an estimation of auditory processing of verbal input within the temporal lobe [53]. It implies the use of various speech stimuli presented simultaneously to both ears. According to Asbjornsen and Hughdahl, attentional instructions lead to enhancement of REA in forced right conditions and enhancement of LEA in forced left conditions [43]. The suppression of intrusive responses in the unattended ear could be explain these results, highlighting the value of controlling for attentional bias. Helland et al. showed that the ability to modulate attention during dichotic listening is weaker in dyslexic children [54]. Such a result is consistent with the findings herein, showing an increase in intrusion errors in the directed or forced recall condition in IR. Furthermore, more intrusive responses were observed for the RE in this population. To be able to repeat successfully in directed recall conditions, one must be able to shift attention to the ear and inhibit the contralateral ear. On one hand, asymmetrical attentional mechanisms may explain REA by a reduced ability to focus attention on the LE. On the other hand, the model proposed by Kinsbourne, which relates to the structural organization of the brain, emphasizes the fact that the processing of verbal material primes the left cerebral hemisphere, while simultaneously inhibiting the right cerebral hemisphere [55]. Since the contralateral afferent auditory pathway is dominant [56], one explanation could be that the signal from the LE progresses to the right hemisphere and decays to the left via the CC, while the signal from the RE transits directly to the left hemisphere to be processed linguistically. Such mechanisms of left cerebral hemisphere priming and right hemisphere inhibition could be deficient in IR.

In humans, efferent projections to the cochlea are constituted by medial olivocochlear system (MOCS) fibers that originate in the superior olivary complex [57]. Its integrity can be assessed by measuring a brainstem reflex mediated by auditory nerve fibers, cochlear nucleus neurons, and efferent fibers to the cochlea [58]. The MOCS is activated by contralateral acoustic stimulation and produces a suppression of cochlear responses. However, the influence of cortical descending pathways in the OC reflex is largely unknown. Indeed, MOCS fibers have been shown to be under cortical control (descending projections from the auditory cortex that are directed towards the thalamus, inferior colliculus, cochlear nucleus, and superior olivary complex) [57,59]. Alterations in the MOCS, affecting language perception in reduced samples of IR, have already been described [60]. It is possible to imagine a role for this system in the pathophysiology of dyslexia—for example, by hypothesizing an alteration of cortical control (possibly by cortical maturation deficits) on the audio-phonatory loop.

Typically, language lateralization develops from more diffuse hemispheric representation in infancy to left hemispheric lateralization in adulthood [27]. Children with impaired language functions may show slower lateralization development compared with typical children, or this development may not take place at all. It is still not known if this atypical development is a cause, correlate, or consequence of language development [19]. DL tasks could reveal incomplete cerebral maturation and lateralization in populations with learning disabilities [37,41,61]. Several studies reported that dyslexic children performed more poorly in their RE than typical readers [44]. The present data are consistent with these results, having shown higher scores on the RE in the forced recall and free recall conditions in non-IR. Younger children are known to have a less pronounced REA, but, parallel to language development, there could be a gradual shift towards the left hemisphere [62,63]. However, some studies even reported a larger REA in IR [64]. The lack of ear advantage is often reported in dyslexia [13,35,44,65], but not in all studies [66]. In the current study, however, no significant difference was found in EP between the groups of non-IR and IR. 

In summary, DL analyses can be complex, as some studies indicate that handedness, age, and sex may interact with DL scores [66,67]. Only right-handed children were included herein, and the sex ratio was similar for each age level in both reading groups. Interestingly, however, in a large-scale study of subjects ranging from age 10 years to older adults, it was concluded that handedness did not affect any of the findings, and no sex differences were seen in children and older adults [68].

## 5. Conclusions

Dichotic listening performances improve with age as the central auditory pathways mature. In IR, at any age, performances remain lower than in non-IR, and the hypothesis of incomplete maturation of the auditory pathways is supported. It seems worthwhile to offer early follow-up to children with reading disorders, by offering them personalized auditory training whenever possible.

## Figures and Tables

**Table 1:** Characteristics of impaired readers and non-impaired readers according to age group.

	Age Groups					
	8–9 y	9–10 y	10–12 y	12–18 y	≥18 y	Total
**Non-IR, *n***	24	24	24	24	24	**120**
Female/male	11/13	11/13	11/13	12/12	11/13	**56/64**
Mean age (SD), months	108 (7.23)	130 (5.73)	147 (5.90)	181 (17.92)	278 (61.41)	
Mean age, years	9	10.8	12.2	15	23	
**IR, *n***	24	24	24	24	24	**120**
Female/male	12/12	12/12	12/12	10/14	11/13	**57/63**
Mean age (SD), months	110 (5.75)	128 (5.11)	145 (5.25)	174 (12.65)	265 (50.08)	
Mean age, years	9.1	10.6	12	14.5	22	

**Table 2:** Mean scores for attended and intrusive responses, attentional shift index and scores in free recall condition for both reading group, each age group and for each ear. Significantly different scores between the left and right ear for each reading group and age group are shown in bold. LE: left ear; RE: right ear; IR: impaired readers; Non-IR: non impaired readers.

	Age (Years)	Ear	Attended Responses	Intrusives Responses	Attentionnal Shift Index	Scores in Free Recall
**Non-IR**	8–9	RE	**89.7**	**18.7**	**2.63**	69.2
		LE	**74.1**	**8.80**	**1.58**	57.8
	9–10	RE	**89.0**	**17.8**	2.70	74.9
		LE	**74.5**	**7.61**	1.69	65.1
	10–12	RE	90.7	11.8	3.01	78.3
		LE	82.5	8.08	2.18	66.0
	12–18	RE	94.8	10.1	**3.46**	81.7
		LE	83.0	4.31	**2.32**	78.7
	≥18	RE	96.1	5.71	**4.20**	83.2
		LE	89.5	2.92	**3.15**	79.9
**IR**	8–9	RE	**73.3**	**32.3**	1.52	52.2
		LE	**56.0**	**18.2**	0.626	39.2
	9–10	RE	**79.3**	**24.2**	1.98	56.1
		LE	**60.6**	**13.7**	0.998	46.4
	10–12	RE	**84.1**	**22.6**	**2.35**	65.4
		LE	**68.9**	**11.2**	**1.22**	52.2
	12–18	RE	87.8	14.7	2.60	67.9
		LE	76.5	7.61	1.78	59.7
	≥18	RE	**90.7**	14.1	3.04	73.5
		LE	**75.2**	7.20	1.83	64.9

## Data Availability

The data presented in this study are available on request from the corresponding author. The data are not publicly available due to ethical, legal and privacy issues.
